# Development of In-Needle SPME Devices for Microextraction Applied to the Quantification of Pesticides in Agricultural Water

**DOI:** 10.3390/molecules29112628

**Published:** 2024-06-03

**Authors:** Ahmed Ali Alshehri, Bechir Hammami, Mohammed Mousa Alshehri, Taieb Aouak, Rabab A. Hakami, Ahmed Yacine Badjah Hadj Ahmed

**Affiliations:** 1 Department of Chemistry, College of Science, Qassim University, Buraidah 51452, Saudi Arabia; b.hammami@qu.edu.sa; 2 Saudi Food and Drug Authority, Riyadh 13513, Saudi Arabia; mmalshehri.ksu@gmail.com; 3 Department of Chemistry, College of Science, King Saud University, Riyadh 11451, Saudi Arabia; taouak@ksu.edu.sa; 4 Department of Chemistry, Faculty of Since, King Khalid University, Abha 61413, Saudi Arabia; raalhakami@kku.edu.sa

**Keywords:** SPME, in-needle coating, PDMS sorbent, GC-MS, pesticides, water

## Abstract

The chemical industry explosion in the 20th century has led to increased environmental pollution, affecting fauna, flora, and waterways. These substances alter water’s taste, color, and smell, making it unfit for consumption or toxic. Agricultural water networks face threats from pollution before and after treatment. Some chemical contaminants, like pesticides, are embedded in natural biogeochemical cycles. In this study, we developed a simple and low-cost procedure for the fabrication of needles coated with polydimethylsiloxane (PDMS) as an efficient sorbent for the microextraction of organic pollutant traces from water. The prepared needles were used as an alternative for commercial solid-phase micro-extraction (SPME) devices in analytical chemistry. The PDMS polymeric phase was characterized by Fourier-transform infrared spectroscopy (FT-IR), thermogravimetry (TGA), and scanning electron microscopy (SEM). The PDMS-coated needles were used for extraction of thirteen pesticides by direct-immersion solid-phase microextraction (DI-SPME) from contaminated waters, followed by determination with gas chromatography-mass spectrometry (GC-MS). The developed analytical method showed limits of detection (LODs) between 0.3 and 2.5 ng mL^−1^ and RSDs in the range of 0.8–12.2%. The homemade needles were applied for the extraction of pesticides in surface and ground aqueous samples collected from an agricultural area. Several target pesticides were identified and quantified in the investigated water samples.

## 1. Introduction

Water is a renewable resource constantly threatened by numerous sources of pollution. Water quality is influenced by various factors, such as precipitation, climate patterns, soil composition, vegetation cover, geological characteristics, flow dynamics, groundwater sources, and anthropogenic activities. In addition, agriculture also has a considerable impact on water quality, due mainly to fertilizers, pesticides, sediments, and inorganic pollutants [1]. Water pollution arises primarily from agricultural and urban runoff that leaches pesticides into the soil, or from the direct discharge of contaminated wastewater [2,3]. The main source of this pollution is agricultural leaching, which results in the transfer of these substances from the soil into groundwater or surface water due to rain and irrigation [4]. Over the past decades, the improvement in agricultural yields due to the increasing use of pesticides has raised concerns about their potential risks to human health and the environment [5,6]. Several studies have investigated the effects of pesticide contact on human health, as well as their potential risks in terms of water pollution, bioaccumulation, air emissions, and soil organisms [7].

The analysis of pesticide residues in water usually involves several stages, mainly including the following: the collection and preparation of samples, extraction, clean-up, separation, and qualitative and quantitative determination. The emergence of green analytical chemistry has led to the development of new extraction methods that have lower human and environmental impacts [8,9,10]. Besides liquid–liquid extraction, which is the conventional procedure used for the isolation of trace pesticides from aqueous samples [11], several other techniques have also been used, including solid-phase extraction, dispersive solid-phase extraction [12], matrix solid-phase extraction [13,14], supported liquid extraction [15], and dispersive liquid–liquid microextraction [16]. Additionally, QuEChERS (quick, easy, cheap, effective, rugged, and safe) is a widely used analytical approach that simplifies the analysis of multiple pesticide residues in fruit, vegetables, cereals, and processed food [17].

The concept of solid-phase microextraction (SPME) was first introduced by Pawliszyn et al. in 1990 [18]. This green technique offers several advantages, such as simplicity, low cost, drastic solvent reduction, and improved accuracy, so it was successfully used in various analytical applications. The original design of the device involved a syringe equipped with a fiber coated with a suitable sorbent phase, and several improvements have been made to this technique [19,20,21,22,23,24]. In addition, SPME provides two different extraction methods: the direct immersion mode (DI-SPME), where the fiber is fully submersed in the aqueous phase, and the headspace mode (HS-SPME), by placing the fiber in the space above the sample [24]. The most common commercially available polymeric coatings are polydimethylsiloxane (PDMS), polyethylene glycol (PEG), and polyacrylate (PA), while various other materials were developed and used for specific applications [25,26,27]. Due to its remarkable properties, PDMS proved to be a versatile and efficient coating for the microextraction of a wide range of trace analytes by SPME [28,29]. In addition to its availability and low cost, PDMS is thermally and chemically stable; additionally, it can be blended with various polymers and materials to prepare specific sorbents [30,31].

In 2016, Rodriguez-Lafuente et al. developed a method for the detection of 25 semi-volatile pesticides in groundwater based on SPME coupled with GC/MS. Four different types of fibers were evaluated in the study, including PDMS (100 µm), PA (85 µm), CAR/PDMS (75 µm), and PDMS/DVB (65 µm), which exhibited the highest extraction efficiency for the investigated pesticides [32]. Another research group has prepared a fiber consisting of a stainless-steel wire coated with a mixture of PDMS and C_18_ particles. This coated wire was used for the extraction of six organo-chlorine pesticides by DI-SPME in water samples, followed by GC-MS measurements [33].

Jabali and his research group have evaluated the occurrence of insecticides in surface waters using microextraction with commercial fibers coated with PDMS (7 µm and 100 µm) and PA (85 µm), the latter being more efficient for extraction of most pesticides. Additionally, a comparison of the two SPME modes revealed that the direct immersion mode (DI-SPME) allowed for the extraction of more target analytes with higher efficiency than the headspace mode (HS-SPME) [34]. The occurrence of 23 pesticides in water sampled from Brazilian cities was studied by do Carmo et al., who developed and optimized a procedure based on DI-SPME using a fiber with a bipolar porous coating [35].

A novel SPME fiber coated with a layer of a highly porous zirconium-based metal-organic framework was developed by Gong et al. for direct immersion extraction of organochlorine pesticides in water samples. This fiber showed better extraction efficiencies than commercial fibers, with 2–20 times higher enrichment factors. Moreover, the prepared coated fiber also exhibited higher extraction efficiencies toward polycyclic aromatic hydrocarbons and nitrobenzenes [36]. For extraction of pesticides from aqueous samples by DI-SPME, Wang et al. have prepared a fiber coated with a material consisting of silicone sealant and a hollow zinc oxide-cerium oxide composite. The high hydrophilicity of SPME sorbents enhances the detection of pesticides when using chromatographic analysis [28,37]. 

A novel stable and crosslinked chitosan/graphene oxide aerogel was prepared and used as a coating for DI-SPME by Peng et al. in 2022. The high porosity and large surface area of this material resulted in high extraction performance towards various hydrophobic pollutants, such as pesticides, polycyclic aromatic hydrocarbons, and polychlorinated biphenyls, in comparison with two commercial fibers [38]. In 2018, Dargahi et al. developed a new SPME device consisting of a platinum wire covered with polypyrrole-zinc oxide nanorods. They used this organic/inorganic nanocomposite wire for microextraction and quantification of pesticides in agricultural water samples by DI-SPME and GC-MS and reached LOD values of about 0.1 ng mL^−1^ [39]. 

A method for the synthesis of carbon nanomaterials was developed by Valenzuela and co-authors by chemical vapor deposition. The carbon nanoparticles were supported on steel threads applied for pre-concentration and extraction of 24 insecticides from water, using both direct immersion and head-space modes. Their results showed low detection limits, with LOQs from 0.0007 to 3.7320 μg L^−1^ [40]. A method for the fabrication of SPME fiber was described by Majdafshar et al. who prepared a polyoxometalate-based ionic liquid that was immobilized onto a stainless-steel wire. This new coated nanomaterial was assessed for the extraction of six triazole pesticides from head-space and aqueous samples, using GC-MS. Excellent repeatability was obtained for the target analytes, while the detection limits were in the range of 7–40 pg mL^−1^ [41]. The concentration of organochlorine pesticides in water was determined by SPME using coatings based on gold nanoparticles developed by Gutiérrez-Serpa et al. in 2017, followed by GC-ECD. Despite the small thickness of the uniform coating, about 3 µm, this homemade needle allowed them to reach detection limits down to 0.13 ng mL^−1^ and extended lifetime. The extraction efficiency of this device was also higher than that of the commercial polyacrylate needle [42].

The interferences caused by the matrix effect on the quantification of pesticides in aquatic matrices by SPME-GC-FID were explored by Silva and his colleagues in 2017. The use of a commercial fiber coated with non-polar PDMS has shown satisfactory results for the extraction of various pesticides. However, the extraction efficiency was lower for more polar pesticides, so the authors suggested the use of SPME fibers coated with less hydrophobic materials [43]. Zang et al. 2021 have fabricated SPME fibers coated with boron nitride-modified multiwalled carbon nanotubes for the extraction of organochlorine pesticides from agricultural samples by using SPME coupled with GC/ECD. Their method proved to be efficient and reliable for determining these residues in fruits and vegetables; it was also compared to three commercial fibers and gave higher extraction performance and reproducibility [44]. A method for the preparation of SPME fibers coated with metal–organic gels was developed by a research group that used a hydrothermal growth technique and optimized the preparation process. This fiber exhibited good extraction efficiency and wide linearity for eleven pesticides, with detection limits between 0.001 and 0.052 ng mL^−1^. These coated fibers proved to be unaffected after 100 consecutive extractions, with the potential for pesticide extractions from complex samples [45]. The present article aims to describe a low-cost and simple method for the preparation of an SPME device by coating silicone sealant onto stainless steel syringe needles. First, the needles used are cheap and commercially available; secondly, the silicone used as a sorbent material is easily purchased from any local market. These simple SPME needles should be a valuable alternative to commercial SPME holders and fibers, which are expensive and fragile. The obtained PDMS-coated needles were characterized, and then applied in the extraction of various pesticides from agricultural water samples by DI-SPME, followed by GC-MS measurements.

## 2. Results and Discussion

### 2.1. Characterization of the Homemade Needle Coated with PDMS

The objective of characterization in this study was to evaluate the properties of the prepared needles coated with PDMS in order to optimize their efficiency and range of application. This investigation was achieved via the utilization of FTIR, TGA, and SEM.

**Figure 1:**
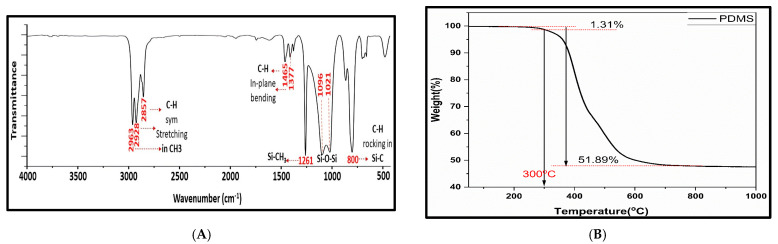
Characterization of needle coated with PDMS by (**A**) FT-IR of the PDMS polymer and (**B**) TGA of the PDMS polymer.

The infrared absorption spectra characteristics of the main functional groups of the prepared PDMS coating were recorded using an FT-IR spectrometer and were compared to those of another PDMS sample from a study carried out by Lee et al. [46]. The main absorption signals corresponding to the different functional groups in PDMS were identified at the following wavenumbers: from 2963 to 2857 cm^−1^ (C–H stretching in CH_3_), 1261 cm^−1^ (CH_3_ symmetric bending in Si–CH_3_), 1096 cm^−1^ and 1021 cm^−1^ (Si–O–Si), and at 800 cm^−1^ (CH_3_ rocking in Si–CH_3_), as shown in Figure 1A.

TGA analysis was performed to further study the thermal stability of the prepared PDMS layer, the decomposition process could be separated into two stages. The first stage occurs in the temperature range from 100 to 300 °C with a weight loss of 1.31%; it mainly corresponds to the elimination of remnant organic matter and humidity. Afterward, the second decomposition stage is observed in the interval from 300 to 600 °C with about 50.58% weight loss; it could be explained by the disintegration of PDMS chains. The results showed that the PDMS polymeric crosslinked material is thermally stable below 300 °C. This is a very important parameter to confirm the thermal stability of the sorbent material (PDMS) for extraction in highly efficient and reproducible applications in microextraction techniques. This can be seen in Figure 1B.

**Figure 2:**
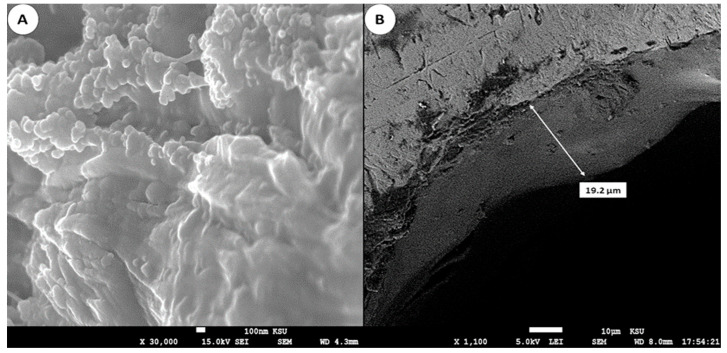
Scanning electron microscopy of the PDMS homemade needle; (**A**) internal surface of the PDMS layer; (**B**) thickness of the PDMS layer.

The morphology of the homemade needle coated with PDMS was evaluated by SEM. As shown in Figure 2A, the PDMS surface appears as a rough and strongly uneven aspect; this morphology increases the possibility of diffusion of the analytes and their transfer from the aqueous phase. Additionally, the presence of wrinkles with pores contributes to increasing the surface area of the sorbent and enhances its extraction capability. In addition, in Figure 2B, the layer thickness on the needle coated with PDMS is unequal, and it was evaluated to be in the range between 10 and 20 µm.

### 2.2. Optimization of SPME-GC-MS Operating Parameters

A standard solution of the selected analytes was injected, and each pesticide was identified and characterized by its retention time and mass spectral data. The optimization of the GC-MS operating conditions involves multiple steps: (a) development of the best method for pesticide chromatographic separation, (b) identification of the highest ions in the mass spectra of target pesticides, and (c) separation of pesticides by SIM mode. The chromatographic selectivity and sensitivity were evaluated for each pesticide.

### 2.3. Optimization of the SPME Extraction Using the Prepared PDMS Needle 

**Figure 3:**
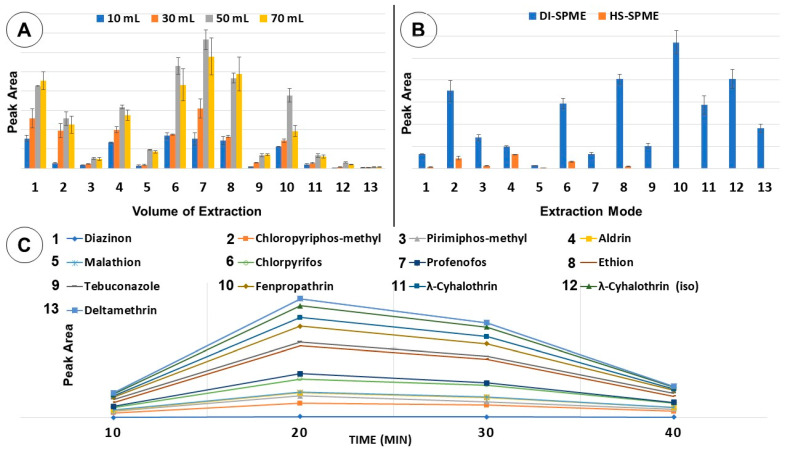
Optimization of SPME extraction: effect of (**A**) volume of extraction; (**B**) extraction mode; (**C**) time of extraction. The peak area is given as a relative value for each pesticide, with the corresponding standard deviation.

The prepared needle coated with PDMS was used for the isolation of pesticides from spiked solutions in order to evaluate and improve its extraction capability by adjusting the main parameters to achieve optimal removal of the target analytes from the aqueous sample by adsorption on the sorbent layer, then desorption into the injection port of the chromatographic system. This evaluation was first conducted on the effect of extraction volume; four distinct volumes of a standard solution of pesticides (with concentrations of 100 ng mL^−1^) were tested: 10, 30, 50, and 70 mL. The intensity of the signal increased from 10 to 50 mL, then diminished at 70 mL for most pesticides. This decrease was attributed to the limited capacity of the needle coating to absorb higher amounts of analyte. Thus, as can be seen in Figure 3A, the best extraction efficiency was achieved for an extraction volume of 50 mL, which corresponded to the highest peak areas. 

In the case of liquid samples, two extraction modes can be used in SPME: direct immersion (DI-SPME) and headspace (HS-SPME). In DI-SPME, the needle is completely immersed in the aqueous phase of the sample, and this is the method of choice for analyzing “clean” aqueous samples, because it is more sensitive. On the other hand, in HS-SPME, the needle is placed in the vapor phase (headspace) above the liquid solution for the extraction of the volatile compounds. The two techniques have been applied and compared; the DI-SPME allowed for the extraction of all the 13 investigated pesticides, and it exhibited greater peak intensities than those obtained by HS-SPME, which could extract only six analytes, as shown in Figure 3B.

In another step, the SPME optimum extraction time was assessed using the homemade PDMS needle in order to enhance the SPME method performance. The following extraction times were evaluated: 10, 20, 30, and 40 min. The results showed that almost all target pesticides reached their extraction equilibrium at 20 min. However, diazinon showed the lowest extraction yield during all the evaluated time ranges. On the other hand, as shown in Figure 3C, most analytes reached the maximum level of extraction at 20 min. Therefore, this extraction time was selected as the optimum value to allow for a faster extraction with better efficiency.

In this study, the influence of stirring the water solution was also evaluated by comparing our results to those of previous studies and selecting the optimal conditions. It was shown that it was possible to reduce the time to achieve equilibrium between the aqueous phase and the needle coating by a thorough agitation of the water sample [47]. On the other hand, the accumulation of the analyte in the polymeric layer is regulated by its diffusion in both the aqueous matrix and the SPME coating, as stated by Arthur et al. [48]. Additionally, in the static situation, the aqueous layer generated on the surface of the sorbent slows down the absorption rate by restricting the transfer of the analyte from the aqueous phase to the solid phase. In contrast, in the dynamic mode (i.e., while being stirred) a thinner layer of water accumulates on the polymer surface, which favors analyte diffusion [48]. Another phenomenon has been observed by Menezes Filho et al. [49] when the aqueous sample was stirred, and the needle immersed in the sample remained immobilized. This stirring mode caused the effect of washing the sorbent surface and induced a reduction in the extraction efficiency of the analytes as the stirring velocities increased. Additionally, another previous study reported that a rapid stirring could reduce the extraction yield because of the air bubble formation on the fiber surface [50]. Based on all previous studies and the results of our experiments, it was concluded that an excessive stirring speed could affect the extraction efficiency, so the suitable stirring rate was chosen to be 250 rpm.

In addition to the TGA results, the thermal stability of the PDMS-coated sorbent inside the needle was assessed by changing the temperature of the injector. The GC inlet temperatures investigated were 250, 300, and 350 °C. Initially, the injection port temperature was set at 250 °C, and good thermal stability of the PDMS needle was observed, as it did not deteriorate in these conditions. At 300 °C, the PDMS coating showed a characteristic degradation which resulted in a series of linear and cyclic siloxane derivatives on the chromatogram, but with a quite low abundance. When using an injector temperature of 350 °C, the PDMS material was severely degraded and the intensity of the siloxane peaks notably increased. These results confirm those obtained in the TGA characterization of the PDMS polymer. Thereupon, it was proven that the PDMS sorbent was thermally stable inside the needle up to 300 degrees and that the needle can be used repeatedly below this temperature.

### 2.4. Method Performance and Validation 

The analytical efficiency of the developed analytical method using SPME based on prepared PDMS needles followed by GC-MS was evaluated to validate its performance. This validation was based on several parameters, such as the linearity coefficient, limits of detection and quantitation (LOD, LOQ), accuracy, and precision, as shown in Table 1. These evaluations were conducted at different levels of concentration to validate the SPME-GC-MS method. The external standard calibration curve was constructed using a series of standard solutions sequentially analyzed in order from low to high concentration. Each concentration was analyzed in triplicate. The linearity of the SPME method was studied by extracting the aqueous solutions spiked with pesticides in concentrations between 0.05 and 1000 ng mL^−1^. The obtained correlation coefficients (R^2^) varied from 0.9990 to 0.9999. On the other side, the limits of detection and quantitation (LOD and LOQ) were calculated using the signal-to-noise ratio for each compound. As is reported in Table 1, the LOD and LOQ values were in the ranges from 0.3 to 2.5 ng mL^−1^ and from 0.9 to 7.5 ng mL^−1^, respectively. 

The precision of the method was evaluated by evaluation of the relative standard deviation (RSD %) of the concentration calculated in intra-day and inter-day experiments, after preparation of a standard solution of all pesticides at a constant concentration (100 ng mL^−1^). The intra-day relative standard deviation (RSD %) value varied from 0.8%, for diazinon, to 12.2%, for λ-cyhalothrin. Furthermore, most of the intra-day RSD values were less than 20%. Additionally, the inter-day RSD values varied from 3.9%, for malathion, to 16.5%, for diazinon. The RSD values of the intraday and inter-day experiments were below 20%, which is acceptable for extraction by SPME.

The relative recoveries were evaluated by extraction and analysis of water samples spiked with a known concentration of the studied pesticides. Two different concentration levels of pesticides were selected, 50 and 250 ng mL^−1^, and each spiked solution was extracted and injected in triplicate. The recovery at the 50 ng mL^−1^ level varied from 77.56%, for deltamethrin, to 116.93%, for chlorpyrifos, while, at 250 ng mL^−1^, the values were between 89.36%, for λ-cyhalothrin, and 116.23%, for deltamethrin. For the studied pesticides, the recoveries for the two concentration levels were between 70 and 120%. These results confirm the good accuracy of the extraction method based on SPME and GC-MS.

### 2.5. Determination of Pesticides in Agricultural Waters

The developed and validated analytical method, using PDMS homemade needles for DI-SPME followed by the GC-MS method, was applied for the investigation of a series of surface and groundwater samples brought from different locations in the Qassim agricultural region (Saudi Arabia). Initially, a quick screening was applied to each sample using the optimized conditions, in order to identify those containing pesticide residues. In a second step, the samples identified as containing pesticide traces were analyzed again in three replicates in order to confirm and quantify the pesticides detected in the previous screening. Among the collected samples, five showed the presence of pesticides, while the remaining waters did not contain any detectable target pesticide. The results of these analyses are summarized in Table 2. Among the target pesticides, seven were identified and quantified in five examined agricultural and irrigation waters. The most contaminated waters were samples S1 and S6 with three and two pesticides, respectively, whereas only one pesticide was found in samples S2, S5, and S7.

### 2.6. Comparison with Previous Studies

The pesticide concentrations shown in Table 2 were compared to results reported in previous studies about the determination of pesticide traces in water samples, as can be seen in Table 3. The selected references described the investigation of pesticide residues in water samples by microextraction techniques followed by GC-MS analysis. The pesticide levels found in the examined samples are in the range of 42.2–155.9 ng mL^−1^. These values are notably higher than those reported for the same pesticides in water samples collected in different regions of the World, which were between 0.069 and 3.52 ng mL^−1^. This significant difference could be explained by the fact that all the surface and groundwater samples examined in the present study were brought from an agricultural area, with a high probability of the presence of pesticides.

On the other hand, a comparison was carried out between some previous studies on pesticide analysis using SPME techniques with the results obtained in the present work with homemade needles coated with PDMS. All these studies highlighted the simplicity and great capacity of the extraction methods based on SPME for the determination of pesticides in various samples. For instance, a study authored by Omena et al. investigated the pesticide residues in rice using a series of commercial SPME fibers. The rice was extracted for 40 min at a temperature of 80 °C, and the desorption time was 10 min. Several pesticides were identified and quantified in rice, including tebuconazole. The validation results showed limits of detection of between 0.46 and 5.9 and RSD% values in the range of 1.3–19% [53]. Similarly, Merib et al. have developed a method for the extraction and determination of pesticides in bovine milk samples by HS-SPME. The extraction time was 90 min, the extraction temperature was 80 °C, and the desorption time was 15 min. They could reach LOD values in the range of 0.2–0.4 ng mL^−1^, while the RSD values were between 2 and 24.6% [54]. Additionally, Wang et al. prepared a fiber including a MOF layer for the extraction and detection of pesticides in fruits; the extraction time was 30 min, and the time taken for the desorption of analytes was 5 min. The method validation gave, for the limits of detection and quantification values, between 0.032 and 0.09 ng mL^−1^ and 0.11–0.30 ng mL^−1^, respectively. Additionally, the relative standard deviation (RSD%) was assessed as between 3.7% and 10.0% [55]. Comparing their optimized parameters with those obtained in the present study, our extraction time was 10 min lower. Additionally, the desorption time for the analytes was 5 min. Thus, the results showed a slight superiority of the homemade PDMS needle, with a less thick coating compared to their MOF fiber. 

The validated method proposed for the SPME microextraction of pesticides from water samples, followed by GC-MS, was compared to the results of previous studies. Table 4 shows a summary of this comparison based on the main validation parameters. It is clear that, for most investigated pesticides, the limit of detection obtained with the presently developed method is significantly higher than those reported in previous articles. However, this difference is particularly noticed when the detection was performed by tandem mass spectrometry, while the LOD results obtained using single quadrupole MS are close to those of the present study. A similar correlation is observed for the linear range, which shows a lower concentration range for the methods based on MS/MS detection.

### 2.7. Comparison of the Pesticide Concentrations in Water with Regulatory Values

The number and amount of pesticides dumped into the environment are continuously increasing, such that the concentrations of pesticide residues found in surface water and groundwater present a serious threat to human health and the environment. Various regulatory limits were established by national, regional, and international institutions for pesticide residues in different kinds of water. Table 5 summarizes the maximum admitted levels of the investigated pesticides in surface, ground, or agricultural waters, or in drinking water. It should be noticed that the regulatory values for drinking water are always lower than those admitted for other water qualities. A comparison of these admitted concentrations to the validation parameters obtained by the developed SPME-GC-MS method shows that the maximum levels of pesticides are higher than the corresponding LOD value. Thus, the analytical method proposed in the present study is suitable for the determination of the investigated pesticide residues in irrigation, agricultural, surface, or groundwater waters. The pesticide concentrations found in the examined water samples were also compared to the regulation values. Except for pirimiphos-methyl, which showed a level lower than its corresponding maximum residue limit, the concentrations of the other detected pesticides notably exceeded the maximum allowed limits. These results are worrying, and confirm the importance of the analysis of irrigation waters, as well as the reasonable use of pesticides.

## 3. Materials and Methods

### 3.1. Chemicals and Materials

The solvents (methanol, acetone, and dichloromethane) were procured from Fisher Scientific (Loughborough, UK). The pesticide standards used in the present work include diazinon, chlorpyrifos-methyl, pirimiphos-methyl, malathion, aldrin, ethion, tebuconazole, profenofos, fenpropathrin, lambda-cyhalothrin, and deltamethrin; they were purchased from Sigma-Aldrich (Steinheim, Germany). Additionally, the commercial silicone sealant for general purpose (PDMS glue) (Wacker, Munich, Germany) was bought from the local market and used for the fabrication of the microextraction needles. The stainless-steel needles used for microextraction were made by Hindustan Syringes & Medical Devices (Haryana, India) and bought in the local market (needles 22 GX1, 0.8 o.d. × 50 mm length). Additionally, for water extraction, we used 50 mL glass vials with septum screw caps from Lemon Vial Company (Quzhou, China). Ultra-pure water was obtained from a Milli-Q water device (with 18.2 MΩs resistivity), which was produced by an Advantage A10 water purification system (Millipore, Molsheim, France).

The stock solution of pesticides was prepared by dissolving 1 mg of each standard pesticide in 10 mL of methanol. Ten samples of surface water and groundwater were collected from the agricultural district of Qassim. All samples were collected in pre-cleaned individual amber glass bottles (250 mL), stored at 4 °C, and then transported to King Saud University for analysis.

### 3.2. Preparation of the Homemade Needle Coated with PDMS

To prepare the homemade needle coated with PDMS, 40 mg of commercial PDMS general purpose (glue) and 4 mL of dichloromethane were mixed in a 10 mL glass vial. 

To coat the needle, it was immersed in the PDMS solution at 15 mm depth for 60 s. The sorbent formed a 30 mm length layer inside the needle. After that, a gentle nitrogen stream was passed through the needle for 30 s to evaporate the solvent and allow the formation of a thin and uniform coating on the inner surface. Additionally, acetone was used to clean the external surface of the needle. Finally, the needle head was plugged with a piece of septum to avoid any carrier gas leak, and it was conditioned for 24 h in the gas chromatograph injector for removal of any remaining volatile chemicals in the sorbent layer. The PDMS polymeric coating was characterized by Fourier transform infrared spectroscopy (FT-IR) using a Spectrum Two FT-IR Spectrometer (Perkin–Elmer Spectrum, Buckinghamshire, UK). The thermal stability was evaluated by thermo-gravimetry with an STA 449 F3 Jupiter device supplied by Netzsch (Selb, Germany). The morphology of the PDMS layer was studied by scanning electron microscopy using an ultra-high-resolution Schottky Field Emission SEM device (JEOL 7610F, Tokyo, Japan).

### 3.3. Evaluation of the SPME-GC-MS Method for Determination of Pesticides

In order to optimize the microextraction procedure using the prepared PDMS needle, the different extraction parameters were investigated. The experimental factors considered in this study were the mode of extraction, the extraction time, and the volume of the aqueous sample. All these parameters showed a great impact on both the sensitivity and selectivity of the developed method. On the other hand, the method was validated by evaluation of the linearity, detection limit, reproducibility, accuracy, and precision. The external standard calibration curve was constructed by using concentrations of the standard solutions, which were in the range of 0.05–1000 ng mL^−1^, and each solution was analyzed in triplicate. All standard solutions were stored at 4 °C in amber glass vials closed with septum screw caps. 

### 3.4. Extraction Procedure

The standard solutions were extracted using the prepared PDMS-coated needle in two modes: direct immersion (DI-SPME) and head-space (HS-SPME). For extraction of the pesticide residues from the standard or real solutions, the prepared needle coated with PDMS was immersed in the aqueous phase for 20 min at room temperature (25 °C). During the extraction, the solution was stirred at a constant speed using a magnetic stirrer. After the extraction, the needle was immediately transferred to the GC injector and desorbed for 5 min at 250 °C. The influence of the aqueous sample volume was also explored by extraction of the spiked solutions with volumes of 10, 30, 50, and 70 mL. The other investigated parameter was the extraction time, measured by repeating the operation at 10, 20, 30, and 40 min.

### 3.5. GC-MS Instrumentation

In the present study, the analytical method was developed such that the sample preparation as well as the chromatographic separation comply with green chemistry principles. Thus, in order to reduce its environmental impact, the sample preparation was miniaturized, and the solvent consumption was minimized by using SPME microextraction. For analysis of complex mixtures, GC offers a solventless and efficient separation, compared to other techniques, and the total run time is shortened [10,32]. All GC-MS measurements were performed on an Agilent 7890A GC coupled to an Agilent 5975-C (inert XL EI/CI MSD) with a single quadrupole mass analyzer (Walnut Creek, CA, USA). The chromatographic separations were carried out using a capillary column HP-5MS UI (Ultra Inert) (30 m × 0.25 mm i.d., 0.25 μm film thickness) coated with a low-polar stationary phase (5% phenyl-methylpolysiloxane). The carrier gas consisted of helium with a purity of 99.999% supplied by Abdullah Hashim Gases. The mass spectrometer was operated in both scan and selected ion monitoring (SIM) modes. Data acquisition and analysis were performed using Chemstation software (version F.01.03.2357).

### 3.6. GC-MS Method Development and Optimization

**Figure 4:**
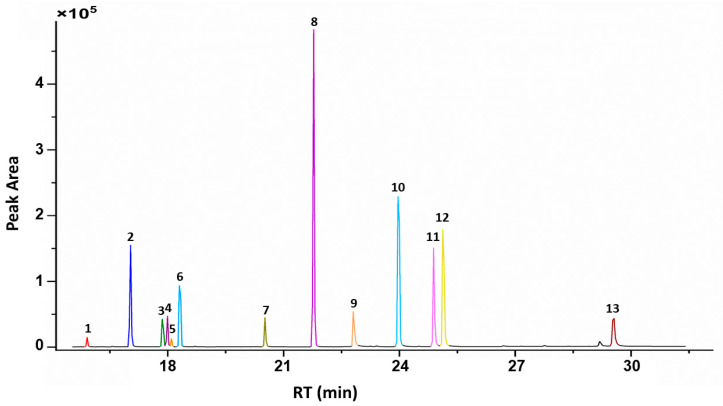
Chromatogram of the target pesticides (1 diazinon, 2 chloropyriphos-methyl, 3 pirimiphos-methyl, 4 aldrin, 5 malathion, 6 chlorpyrifos, 7 profenofos, 8 ethion, 9 tebuconazole, 10 fenpropathrin, 11 λ-cyhalothrin, 12 λ-cyhalothrin (isomer), and 13 deltamethrin).

All chromatographic parameters were explored in order to obtain the best separation and detection of the target pesticides. For GC operation, the optimized conditions were as follows: the injector was heated at 250 °C, and it was operated in split-less mode. Helium was used as a carrier gas at a flow rate of 2 mL min^−1^. The GC oven temperature program was as follows: initial temperature: 75 °C (hold 2 min), rate: 8 °C/min, final temperature: 280 °C (hold 8 min). The complete analysis time was 36 min for all analytes. The chromatogram of a standard solution spiked with the target pesticides is shown in Figure 4.

In the mass spectrometer, the transfer line temperature was set to 250 °C, and the ion source was heated to 200 °C. For sample ionization by electron impact, the electron energy was 70 eV, and the filament current was 50 µA. The first detection mode used was a full scan in the range from 50 to 500 Da. Subsequently, in order to enhance the selectivity and sensitivity, the target pesticides were detected and quantified using the SIM mode. The selected ions and the corresponding retention times are given in Table 6.

## 4. Conclusions

In this work, a straightforward, solvent-free, and relatively rapid analytical methodology based on DI-SPME followed by GC-MS was developed, optimized, and validated to determine pesticide residues in water samples. The microextraction procedure was based on SPME devices fabricated with common commercial stainless-steel needles, which were internally coated with silicone sealant. Although the preparation was simple, rapid, and used low-cost materials, the obtained SPME needles proved to be stable, reusable, efficient, and robust, compared with commercial devices that are based on fragile fused silica fibers. Moreover, PDMS is a hydrophobic sorbent, but the proposed preparation technique could be applied to more polar polymers or composites. The developed analytical method was selective, sensitive, precise, and linear over a wide range of concentrations for the determination of pesticide residues in aqueous media. The investigated pesticides were from different chemical groups (organophosphates, organochlorines, pyrethroids, carbamates, and triazines). The sample preparation procedure involved a solvent-less technique, which provided a simple, reliable, and efficient alternative to traditional methods used for the determination of pesticide residues. The results have shown that the optimized analytical method was adequate for the qualitative and quantitative analysis of pesticides in various water samples with low waste generation and reduced risks to the analyst. The results obtained from 10 water samples extracted with the prepared needle indicated that some of the investigated samples contained pesticides at low concentrations.

## Figures and Tables

**Table 1:** Main validation parameters obtained using the prepared in-needle SPME.

Pesticide	Linear Equation	R^2^	Linear Range (ng mL^−1^)	LOD (ng mL^−1^)	LOQ (ng mL^−1^)	Precision RSD % *		Recovery %			
						Intra-Day	Inter-Day	50 ng mL^−1^	RSD %	250 ng mL^−1^	RSD %
**Diazinon**	y = 1760x – 17,390	0.9998	5–1000	2.1	6.3	0.8	16.5	102.55	19.5	104.90	12.5
**Chloropyriphos-methyl**	y = 11,869x – 102,442	0.9997	2.5–500	1.4	4.3	10.3	13.5	95.63	17.8	109.16	17.5
**Pirimiphos-methyl**	y = 4958x – 22,322	0.9993	2.5–250	2.3	6.9	7.4	9.5	114.03	4.0	95.35	8.0
**Aldrin**	y = 3044.9x + 83,228	0.9996	2.5–500	1.9	5.6	2.9	12.4	78.30	2.8	98.06	6.3
**Malathion**	y = 773.6x – 38,011	0.9997	2.5–500	2.1	6.2	3.1	3.9	103.94	15.7	93.49	8.1
**Chlorpyrifos**	y = 8244.4x + 196,208	0.9992	2.5–250	1.1	3.2	1.6	8.1	116.93	1.7	104.90	9.7
**Profenofos**	y = 2906.8x – 30,920	0.9990	1–500	0.8	2.3	7.6	15.2	82.22	14.4	95.34	4.4
**Ethion**	y = 14,872x – 30,223	0.9998	0.5–250	0.3	0.9	5.3	10.3	103.99	9.2	89.76	6.6
**Tebuconazole**	y = 4404.9x + 39,488	0.9999	1–500	0.7	2.0	11.3	16.0	98.06	8.4	90.54	8.4
**Fenpropathrin**	y = 15,770x – 215,106	0.9996	2.5–500	2.5	7.6	9.4	14.4	109.24	3.5	99.91	1.4
**λ-Cyhalothrin**	y = 4978.6x – 110,934	0.9991	1–500	1.0	3.1	12.2	14.3	104.72	15.5	89.36	7.3
**λ-Cyhalothrin (iso)**	y = 6702.9x – 125,201	0.9991	1–250	0.7	2.2	5.9	10.5	108.14	17.0	104.51	6.6
**Deltamethrin**	y = 1641.1x − 7079.5	0.9993	2.5–500	2.5	7.5	10.7	13.6	77.56	13.2	116.23	4.9

* At a concentration of 100 ng mL^−1^.

**Table 2:** Concentration and detection ranges of target pesticides in 10 water samples.

Pesticide	Water Samples (ng mL^−1^)									
	S1	S2	S3	S4	S5	S6	S7	S8	S9	S10
**Diazinon**	ND	ND	ND	ND	ND	ND	ND	ND	ND	ND
**Chloropyriphos-methyl**	ND	ND	ND	ND	ND	ND	ND	ND	ND	ND
**Pirimiphos-methyl**	42.2	ND	ND	ND	ND	ND	ND	ND	ND	ND
**Aldrin**	155.9	ND	ND	ND	ND	ND	ND	ND	ND	ND
**Malathion**	ND	ND	ND	ND	ND	ND	ND	ND	ND	ND
**Chlorpyrifos**	ND	ND	ND	ND	ND	ND	ND	ND	ND	ND
**Profenofos**	ND	ND	ND	ND	ND	ND	ND	ND	ND	ND
**Ethion**	80.1	87.9	ND	ND	ND	ND	ND	ND	ND	ND
**Tebuconazole**	ND	ND	ND	ND	ND	ND	85	ND	ND	ND
**Fenpropathrin**	ND	ND	ND	ND	ND	132.4	ND	ND	ND	ND
**λ-Cyhalothrin**	ND	ND	ND	ND	ND	ND	ND	ND	ND	ND
**λ-Cyhalothrin (iso)**	ND	ND	ND	ND	114.8	ND	ND	ND	ND	ND
**Deltamethrin**	ND	ND	ND	ND	ND	68.9	ND	ND	ND	ND

ND: not detected pesticides (concentration ˂ LOD).

**Table 3:** Comparison of real sample concentration with previous studies.

Pesticides	Present Work		Previous Studies		
	Concentration (ng mL^−1^)	Sample	Concentration (ng mL^−1^)	Water Type	Ref.
Pirimiphos-methyl	42.2	S1	0.069–0.230	River	[34]
Aldrin	155.9	S1	0.257 3.52	River Well	[51] [52]
Ethion	80.1 87.9	S1 S2	0.14	Surface	[34]
λ-Cyhalothrin	114.8	S5	0.16	Well	[34]

**Table 4:** Comparison of the developed method with those of previous studies.

Pesticides	Present Work			Previous Studies				
	LOD (ng mL^−1^)	Range (ng mL^−1^)	Recovery (%)	LOD (ng mL^−1^)	Range (ng mL^−1^)	Recovery (%)	Detection	Ref.
**Diazinon**	2.1	5–1000	103–105	0.003	0.05–1	85–115	MS/MS	[56]
**Chloropyriphos-methyl**	1.4	2.5–500	96–109	0.01	0.05–50	87–109	MS/MS	[34]
**Pirimiphos-methyl**	2.3	2.5–250	95–114	0.01	0.05–50	80–99	MS/MS	[34]
**Aldrin**	1.9	2.5–500	78–98	1.91	-	44–125	MS	[51]
**Malathion**	2.1	2.5–500	93–104	0.38	0.4–30	81–92	MS	[57]
**Chlorpyrifos**	1.1	2.5–250	105–117	0.002	0.05–1	93–102	MS/MS	[56]
**Profenofos**	0.8	1–500	82–95	0.056	0.17–200	90–101	MS	[58]
**Ethion**	0.3	0.5–250	90–104	0.13	0.4–35	-	MS	[57]
**Tebuconazole**	0.7	1–500	90–98	0.02	0.05–5	102–112	MS/MS	[34]
**Fenpropathrin**	2.5	2.5–500	99–109	-	-	-	-	-
**λ-Cyhalothrin**	1.0	1–500	89–105	0.02	0.05–5	101–105	MS/MS	[34]
**Deltamethrin**	2.0	2.5–500	78–116	-	-	-	-	-

**Table 5:** Regulation values for target pesticides in water.

Pesticides	Water Type	Maximum Values (ng mL^−1^)	Ref.
Diazinon	Ground	100	[59]
	Drinking	15	[60]
Chloropyriphos-methyl	Agricultural	8	[61]
Pirimiphos-methyl	Surface	100	[62]
Aldrin	Surface	100	[62]
Malathion	Ground	500	[59]
	Agricultural	290	[63]
Chlorpyrifos	Ground	100	[59]
	Agricultural	90	[63]
Ethion	Surface	25	[61]
Fenpropathrin	Surface	10.3	[64]
λ-Cyhalothrin	Ground	5.35	[65]
Deltamethrin	Drinking	2.5	[61]

**Table 6:** Retention times in GC and characteristic monitored ions for SIM detection of the target pesticides.

N	Pesticides	M.W	SIM Ions	RT (min)
**1**	Diazinon	304	137, 179, 152	15.790
**2**	Chloropyriphos-methyl	321	286, 288, 125	16.926
**3**	Pirimiphos-methyl	305	290, 305, 276	17.769
**4**	Aldrin	362	263, 265, 261	17.866
**5**	Malathion	330	125, 173, 127	17.996
**6**	Chlorpyrifos	349	197, 199, 314	18.223
**7**	Profenofos	372	139, 208, 206	20.428
**8**	Ethion	384	231, 153, 125	21.693
**9**	Tebuconazole	307	125, 250, 252	22.731
**10**	Fenpropathrin	349	181, 125, 265	23.899
**11**	λ-Cyhalothrin	449	181, 197, 208	24.774
**12**	λ-Cyhalothrin (isomer)	449	181, 197, 208	25.034
**13**	Deltamethrin	503	181, 253, 251	29.380

## Data Availability

The original contributions presented in this study are included in the article, further inquiries can be directed to the corresponding author.
